# Supplementary material for: Simple and Equipment-Free Paper-Based Device for Determination of Mercury in Contaminated Soil
Source: Molecules. 2021 Apr 1;26(7):2004. doi: 10.3390/molecules26072004 (PMC8037038; doi:10.3390/molecules26072004)
Supplement: Supplementary file 1 [file molecules-26-02004-s001.pdf]

Supplementary Information

Simple and Equipment-Free Paper-Based Device for Determination of Mercury in Contaminated Soil

Hikmanita Lisan Nashukha [1,2], Jirayu Sitanurak [1,2], Hermin Sulistyarti [3] and Duangjai Nacapricha [1,2] and Kanchana Uraisin [1,2,*]

1 Flow Innovation-Research for Science and Technology Laboratories (Firstlabs), Mahidol University Rama 6 Road, Bangkok 10400, Thailand; hikmanitalisan@gmail.com (H.L.N.); jsitanurak@gmail.com (J.S.); dnacapricha@gmail.com (D.N.)

2 Department of Chemistry and Center of Excellence for Innovation in Chemistry, Faculty of Science, Mahidol University, Rama 6 Road, Bangkok 10400, Thailand

3 Department of Chemistry, Faculty of Science, Brawijaya University, Jl. Veteran Malang, 65145, Indonesia; sulistyarti@yahoo.com

* Correspondence: kanchana.ura@mahidol.ac.th; Tel.: +66-2-201-5125

Table of Contents

Part 1:

Method S1	Preliminary test for selection of the color scale	2
Figure S1	Calibration graphs obtained from preliminary tests using the iodine-starch reaction on a single-layer paper device with the ImageJ analysis of the plot between (a) grey intensity, (b) red intensity, (c) green intensity and (d) blue intensity against the concentration of iodide.	2

Part 2

Method S2	Control of light illumination and calibration of camera	3
Figure S2	Effect of pH of standard 150 mg L^{-1} Hg solutions on the green intensity value. Experimental conditions: 0.2 mol L^{-1} KIO_3 in 0.2 mol L^{-1} H_2SO_4, 10 mmol L^{-1} KI, 1% (w/v) of starch in 0.1 mmol L^{-1} KI and reaction time of 4 min.	4
Figure S3	Examples of photographic images of the colored product formed in the acceptor reservoirs during analysis of soil and water samples. Note: Soil samples S1-S3 were highly contaminated with mercury whereas the levels of mercury in other samples of soils and waters are below the detection limit.	5

Part 1

Method S1: Preliminary test for selection of the color scale

Preliminary experiments were carried out using a single-layer paper device. This device was produced with the same pattern as the "acceptor layer A" of the μPAD (see Fig. 1a(ii)). Reagent A (2 μL of a solution mixture of 0.2 mol L^{-1} IO_3^- and 0.2 mol L^{-1} H^+) was first dispensed onto the circular reservoir and allowed to dry for 5 min before dispensing a series of I^--starch solution with varying concentrations of I^- (2-9 mmol L^{-1}) but fixed starch at 1 %(w/v). After dispensing the I^--starch solution, the circular reservoir turned from white to purple color. The intensity of the purple color complex of I_3^--starch formed on the reservoir corresponds to the concentration of I^- in the mixture. After recording the images, the image was analyzed using ImageJ (version v1.35e) to obtain the intensity values of grey- and RBG-scales and to plot the calibration graphs. The preliminary results in Figure S1 show that the plot obtained using the green intensity values of the RGB-scales gave the highest sensitivity with r^2 close to 1. (The green color corresponds to the color absorbed when the color observed is purple,) We therefore used the green intensity values for the calibration plots of the 3-layer PADs for this work.

.

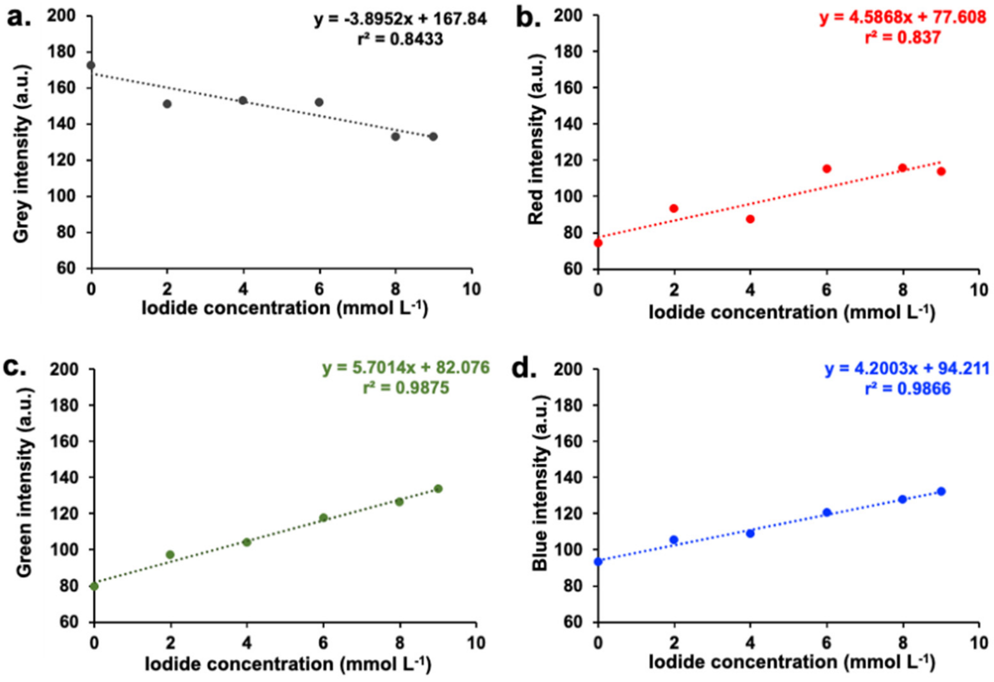

Figure S1. Calibration graphs obtained from preliminary tests using the iodine-starch reaction on a single-layer paper device with the ImageJ analysis of the plot between (a) grey intensity, (b) red intensity, (c) green intensity and (d) blue intensity against the concentration of iodide.

Part 2

Method S2: Control of light illumination and calibration of camera

On the stage for placing the μPAD for photography, a red circular zone (6 mm diameter) was employed to calibrate the camera light sensing system before recording the images of the μPAD. The following is the protocol for recording the image of the acceptor reservoir ‘ar’.

Step 1: The camera (Canon IXUS 125 HS, Japan) is first set to ISO 200. The shutter button is pressed halfway until the focusing frame appears covering the red circular reference zone. The values of ISO 200, speed shutter 1/80 and f-number (aperture) 5.0 are then displayed.

Step 2: Move the focusing frame to cover the area of the acceptor reservoir ‘ar’ and record the image of the purple-colored zone.

Step3: The obtained image is exported to a computer for analysis by ImageJ.

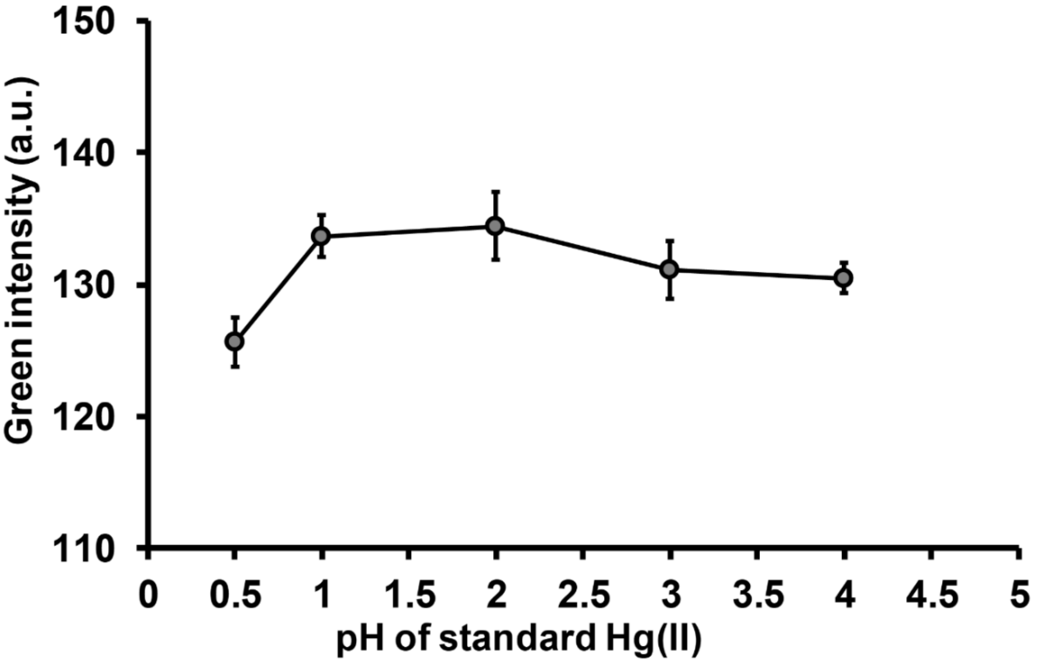

Figure S2. Effect of pH of standard 150 mg L^{-1} Hg solutions on the green intensity value. Experimental conditions: 0.2 mol L^{-1} KIO_3 in 0.2 mol L^{-1} H_2SO_4, 10 mmol L^{-1} KI, 1% (w/v) of starch in 0.1 mmol L^{-1} KI and reaction time of 4 min.

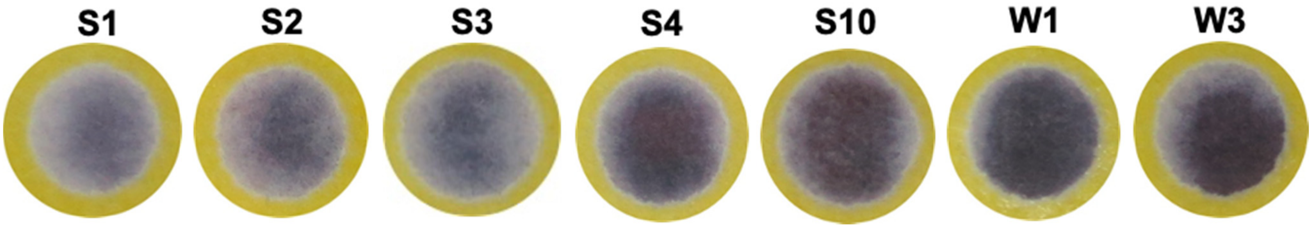

Figure S3. Examples of photographic images of the colored product formed in the acceptor reservoirs during analysis of soil and water samples. Note: Soil samples S1-S3 were highly contaminated with mercury whereas the levels of mercury in other samples of soils and waters are below the detection limit.
